# Life-sustaining treatment preferences in older patients when referred to the emergency department for acute geriatric assessment: a descriptive study in a Dutch hospital

**DOI:** 10.1186/s12877-020-02002-y

**Published:** 2021-01-14

**Authors:** Daisy J. M. Ermers, Marit P. H. van Beuningen-van Wijk, Evi Peters Rit, Sonja C. Stalpers-Konijnenburg, Diana G. Taekema, Frank H. Bosch, Yvonne Engels, Patricia J. W. B. van Mierlo

**Affiliations:** 1 Department of Anesthesiology, Pain and Palliative Medicine, Radboud university medical center, PO Box 9101, Nijmegen, HB 6500 The Netherlands; 2 Department of Pulmonology, Rijnstate, Arnhem, The Netherlands; 3 Department of Geriatrics, Meander medical center, Amersfoort, The Netherlands; 4 Department of Geriatrics, Rijnstate, Arnhem, The Netherlands; 5 Department of Intensive Care Medicine, Rijnstate, Arnhem, The Netherlands; 6 Department of Internal Medicine, Radboud university medical center, Nijmegen, The Netherlands; 7 Center of Supportive and Palliative Care, Rijnstate, Arnhem, The Netherlands

**Keywords:** Advance care planning, Palliative care, Emergency department, Geriatric assessment, Referral, Preferences regarding life-sustaining treatment

## Abstract

**Background:**

In many cases, life-sustaining treatment preferences are not timely discussed with older patients. Advance care planning (ACP) offers medical professionals an opportunity to discuss patients’ preferences. We assessed how often these preferences were known when older patients were referred to the emergency department (ED) for an acute geriatric assessment.

**Methods:**

We conducted a descriptive study on patients referred to the ED for an acute geriatric assessment in a Dutch hospital. Patients were referred by general practitioners (GPs), or in the case of nursing home residents, by elderly care physicians. The referring physician was asked if preferences regarding life-sustaining treatments were known. The primary outcome was the number of patients for whom preferences were known. Secondary outcomes included which preferences, and which variables predict known preferences.

**Results:**

Between 2015 and 2017, 348 patients were included in our study. At least one preference regarding life-sustaining treatments was known at referral in 45.4% (158/348) cases. In these cases, cardiopulmonary resuscitation (CPR) policy was always included. Preferences regarding invasive ventilation policy and ICU admission were known in 17% (59/348) and 10.3% (36/348) of the cases respectively. Known preferences were more frequent in cases referred by the elderly care physician than the GP (*P* < 0.001).

**Conclusions:**

In less than half the patients, at least one preference regarding life-sustaining treatments was known at the time of referral to the ED for an acute geriatric assessment; in most cases it concerned CPR policy. We recommend optimizing ACP conversations in a non-acute setting to provide more appropriate, desired, and personalized care to older patients referred to the ED.

**Supplementary Information:**

The online version contains supplementary material available at 10.1186/s12877-020-02002-y.

## Background

An unplanned hospitalization necessitates in-the-moment decision making. Advance care planning (ACP) recognizes that advance directives (ADs) play an important role and can be used at these decision making moments [1, 2]. ADs include preferences regarding life-sustaining treatments, for example interventions like cardiopulmonary resuscitation (CPR), which need to be considered or undertaken in case of an emergency [3].

ACP can either prevent unnecessary life-prolonging treatment [4] or undertreatment based on ageism. ACP is particularly recommended when individual health conditions worsen, especially in the case of older people [2] where the decisional capacity may decline at some stage [5, 6]. Consequently, ACP should be discussed timely with older people when decisional capacity still exists; an emergency is not an ideal situation to discuss ACP [7]. Therefore, it is recommended that primary care physicians with longstanding doctor-patient relationships [8–10] timely discuss ACP with their patients [11].

In the Netherlands every citizen is registered with a general practitioner (GP); in nursing homes elderly care physicians take on this generalist role [12]. Both GPs and elderly care physicians are gatekeepers for hospital and specialist care. When a patient needs to be referred to the emergency department (ED), the GP or elderly care physician commonly contacts the relevant medical specialist by phone. For an acute geriatric assessment, they usually contact the geriatrician.

Although both GPs and elderly care physicians are aware of the relevance of having ACP conversations, treatment preferences often remain unknown for many individuals at the end of their lives [13, 14] or at the moment of referral to the ED.

We therefore initiated this study to assess how often and which preferences regarding life-sustaining treatments of older patients were known by the referring GP or elderly care physician at the time of referral to the ED for an acute geriatric assessment.

## Methods

### Study design

We performed a descriptive study at a clinical teaching hospital, Rijnstate, Arnhem, The Netherlands, a member of the association of tertiary teaching hospitals.

### Study setting and participants

From June 2015 to January 2017, we included patients referred by the GP or elderly care physician to the ED for an acute geriatric assessment by an on-call geriatrician (geriatrician, geriatric intern or geriatric resident). The assessment is based on the principals of the comprehensive geriatric assessment (CGA) [15] which is used to examine (frail) older people with multimorbidity.

In the Netherlands, GPs and elderly care physicians maintain their own medical record system, which is not exchangeable with hospital medical records. Discussing and documenting life-sustaining treatment preferences is recommended in both primary and hospital care [16, 17]. These are based on recommendations for life-sustaining treatments defined in the Oxford Textbook of Palliative Medicine [18] and include cardiopulmonary resuscitation policy (CPR), admission to the intensive care unit (ICU) or coronary care unit (CCU), invasive ventilation, dialysis, defibrillation, and preferences regarding blood transfusion and antibiotics, or comfort-focused care. Conversations about these preferences mostly concern shared decision making; they are not one-sided physician decisions.

### Study procedure

In May 2015, we discussed the study aim and procedure at a meeting with all geriatricians working at the hospital’s geriatric department. We instructed them to ask each physician referring a patient to the ED for an acute geriatric assessment whether the patient’s preferences regarding life-sustaining treatments were known. In cases where the referring physician was unaware of the preferences, they were asked to check whether these preferences were documented in the medical records or were known by the legal representative if he/she was present. To guide geriatricians when asking for known preferences, they were instructed to use the standard format in the patient’s electronic medical record where physicians fill in treatment limitations regarding life-sustaining treatments. The answers were then documented in the hospital electronic medical record. For the duration of the study, the researchers regularly reminded the geriatricians of the procedure.

### Data collection

Patient characteristics at the time of referral to the ED were collected. These included age, sex, living situation, mobility, presence of a cognitive disorder (evident from a diagnosis or reported by the referrer), Charlson comorbidity index (CCI) [19, 20], main reason for referral, and number of prescriptions. We also collected information regarding the number of hospitalizations in the year prior to the ED visit, as well as mortality during the ED visit or during the subsequent hospitalization.

The primary outcome was whether at least one preference regarding life-sustaining treatments was known. Secondary outcome measures included which preferences were known. Additionally, we noted the type of referrer (elderly care physician or GP), and analysed and corrected for the following patient-specific variables: age, sex, reason for referral, presence of a cognitive disorder, multimorbidity (CCI), mobility, use of home care, number of prescriptions, number of hospitalizations 1 year prior to the ED visit, and mortality during the ED visit or during the subsequent hospitalization. The medical records were reviewed retrospectively to collect data regarding the variables. Data were transcribed into Microsoft Excel and exported to SPSS. All data were anonymized.

### Ethics

At the Rijnstate hospital, all patients are informed that routine registration data are anonymized and can be used for scientific research; they may choose to opt out. This study was performed following the Good Clinical Practice guidelines and the Dutch law (*Wet op de Geneeskundige Behandelingsovereenkomst WGBO* [21] and *Wet Maatschappelijke Ondersteuning WMO* [22]). Therefore, no permission of the medical ethical committee was required, including the need for written informed consent.

### Statistical analyses

Statistical analyses were performed using IBM SPSS Statistics 24. We used descriptive statistics for all outcomes. We applied backward logistic regression to analyse the relation between patient and demographic characteristics, type of referrer and preferences being known. As independent variables, we used type of referrer and the following patient specific variables: age, sex, living situation, mobility, presence of a cognitive disorder, CCI, number of prescriptions, reason for referral and number of hospitalizations in the year before ED visit. As dependent variables, we used preferences being known. The criterion to stay in the model was *P* < 0.2. We only present results for the final model of the backward logistic regression analysis.

## Results

### Study sample

Between May 2015 and January 2017, GPs and elderly care physicians referred a total of 501 patients to the ED for an acute geriatric assessment. In 348 cases (69.5%), the on-call geriatrician asked and documented patient’ preferences and data were collected. Of these patients, 69 (19.8%) were referred by elderly care physicians and 279 (80.2%) by GPs.

### Patient and demographic characteristics at time of referral to the ED

Table 1 shows patient characteristics at time of referral to the ED. Mean age was 84 years, and more than half of the patients were women (61.8%; 215/348). Of all patients, 19.8% (69/348) lived in a nursing home. The other 80.2% (279/348) lived at home, of whom 63.1% (176/279) depended on home care or domestic services. Cognitive disorder was present in 60.6% (211/348) of the cases. For 243 (69.8%) patients, it was their first ED visit that year. Mortality during the ED visit or during the subsequent hospitalization was 6.9% (24/348).

**Table 1:** Patient characteristics at time of referral, reason of referral and mortality during the ED visit or during the subsequent hospitalization (*N* = 348), N (%)

	***Referred by elderly care physician, N = 69***	***Referred by GP, N = 279***	***Total, N = 348***
***Age, per category***			
*60–74*	10 (14.5)	22 (7.9)	32 (9.2)
*75–84*	26 (37.7)	110 (39.4)	136 (39.1)
*85–94*	28 (40.6)	135 (48.4)	163 (46.8)
*≥ 95 years*	5 (7.2)	12 (4.3)	17 (4.9)
***Sex***			
*Female*	43 (62.3)	172 (61.6)	215 (61.8)
***Living situation***			
*Nursing home*	69 (100)	–	69 (19.8)
*At home, with:*	–	279 (100)	–
*- No home care services*	–	99 (35.5)	99 (28.4)
*- Home care services*	–	176 (63.1)	176 (50.6)
*- Unknown amount of services*	–	4 (1.4)	4 (1.1)
***Mobility***			
*Walking*			
*- Independent*	4 (5.8)	66 (23.7)	70 (20.1)
*- With walking aid (walking stick/wheeled walker)*	32 (46.4)	193 (69.2)	225 (64.7)
*Wheelchair dependent*	31 (44.9)	14 (5.0)	45 (12.9)
*Unknown*	2 (2.9)	6 (2.2)	8 (2.3)
***Presence of a cognitive disorder***			
*Yes*	60 (87.0)	151 (54.1)	211 (60.6)
*No*	6 (8.7)	125 (44.8)	131 (37.6)
*Unknown*	3 (4.3)	3 (1.1)	6 (1.7)
***Charlson comorbidity index (CCI)***			
0	1 (1.4)	36 (12.9)	37 (10.6)
1–2	32 (46.4)	160 (57.3)	192 (55.2)
≥ 3	36 (52.2)	81 (29.0)	117 (33.6)
Unknown	0 (0.0)	2 (0.7)	2 (0.6)
***Number of prescriptions***			
0	0 (0.0)	5 (1.8)	5 (1.4)
1–5	3 (4.3)	78 (28.0)	81 (23.3)
6–10	22 (31.9)	114 (40.9)	136 (39.1)
11–15	28 (40.6)	63 (22.6)	91 (26.1)
> 15	14 (20.3)	18 (6.5)	32 (9.2)
Unknown	2 (2.9)	1 (0.4)	3 (0.9)
***Category of the main reason for referral***			
*Internal medicine*	31 (44.9)	105 (37.6)	136 (39.1)
*Acute cognitive decline*	17 (24.6)	92 (33.0)	109 (31.3)
*Functional decline*	3 (4.3)	46 (16.5)	49 (14.1)
*Pulmonological*	5 (7.2)	11 (3.9)	16 (4.6)
*Neurological*	5 (7.2)	10 (3.6)	15 (4.3)
*Psychiatric*	3 (4.3)	6 (2.2)	9 (2.6)
*Chronic cognitive decline*	3 (4.3)	4 (1.4)	7 (2.0)
*Cardiological*	2 (2.9)	5 (1.8)	7 (2.0)
***Number of hospitalizations during one year before ED visit***			
0	48 (69.6)	195 (69.9)	243 (69.8)
1	13 (18.8)	50 (17.9)	63 (18.1)
≥ 2	8 (11.6)	34 (12.2)	42 (12.1)
***Mortality during ED visit or during the subsequent hospitalization***	7 (10.1)	17 (6.1)	24 (6.9)

When comparing patients referred by the elderly care physician to those referred by GPs: walked independently 5.8% (4/69) vs. 23.7% (66/279); cognitive disorder present 87.0% (60/69) vs. 54.1% (151/279); CCI ≥ 3 52.2% (36/69) vs. 29.0% (81/279); > 5 prescriptions 92.8% (64/69) vs. 69.8% (195/279) – all respectively.

These parameters show that our study population generally consisted of frail older people, those referred by the elderly care physician were even more frail.

### Preferences regarding life-sustaining treatments

Preferences regarding life-sustaining treatments were known by the referring physician in 45.4% (158/348) of all patients. With regard to physician type, preferences were known in 88.4% (61/69) of patients referred by the elderly care physician, and in 34.8% (97/279) of patients referred by the GP. When preferences were known, they always included CPR policy (158/158), see Table 2. Of these 158 patients, invasive ventilation preferences were known in 59 cases (37.3%), and ICU admission in 36 cases (22.8%). Other preferences were rarely known (0.6–8.9%). For patients referred by an elderly care physician, preferences regarding invasive ventilation and ICU admission were more often known compared to GP referrals; 44.3% (27/61) vs. 33.0% (32/97), and 27.9% (17/61) vs. 19.6% (19/97) respectively.

**Table 2:** Known patients’ preferences regarding life-sustaining treatments at referral to the ED for an acute geriatric assessment. The study population is split by type of referrer. For patients for whom preferences were known, the frequencies of the individual preferences are presented

	***Referred by elderly care physician, N = 69***	***Referred by GP, N = 279***	***Total, N = 348***
***Patients for whom at least one preference is known; N (%)***	61 (88.4)	97 (34.8)	158 (45.4)
	***Referred by elderly care physician, N = 61***	***Referred by GP, N = 97***	***Total, N = 158***
***Cardiopulmonary resuscitation (CPR) known; N (%)***	61 (100)	97 (100)	158 (100)
*When known, preference ‘yes’; N*	4	10	14
***Invasive ventilation known; N (%)***	27 (44.3)	32 (33.0)	59 (37.3)
*When known, preference ‘yes’; N*	3	7	10
***Admission to the intensive care unit (ICU) known; N (%)***	17 (27.9)	19 (19.6)	36 (22.8)
*When known, preference ‘yes’; N*	3	7	10
***Admission to the coronary care unit (CCU) known; N (%)***	6 (9.8)	8 (8.2)	14 (8.9)
*When known, preference ‘yes’; N*	2	7	9
***Dialysis known; N (%)***	3 (4.9)	6 (6.2)	(5.7)
*When known, preference ‘yes’; N*	2	6	8
***Defibrillation known; N (%)***	3 (4.9)	6 (6.2)	9 (5.7)
*When known, preference ‘yes’; N*	2	6	8
***Only comfort-focused care (no life-prolonging treatments) known; N (%)***	2 (3.3)	4 (4.1)	6 (3.8)
*When known, preference ‘yes’; N*	0	3	3
***Other preferences, including blood transfusion and antibiotics known; N (%)***	1 (1.6)	0 (0.0)-	1 (0.6)
*When known, preference ‘yes’; N*	0	–	0

### Factors related to known preferences regarding life-sustaining treatments

Table 3 shows the results of the final model of the backward logistic regression analysis for factors related to known preferences. Preferences were less frequently known for GP-referred patients (OR 0.075, *P* < 0.001) and for patients aged between 60 and 74 (OR 0.171, *P* < 0.05). Preferences were more frequently known for wheelchair-dependent patients (OR 3.02, *P <* 0.05), those with a cognitive disorder (OR 1.96, *P <* 0.05), and those hospitalized in the year before the ED visit (OR 2.48, *P <* 0.05). Supplementary Table 1 shows the known preferences stratified by type of referrer, and full patient and demographic characteristics.

**Table 3:** End model of backward logistic regression analysis for factors related to preferences being known

Factor	OR (95% CI)	***P***-value
*Type of referrer*		
GP vs. elderly care physician	0.075 (0.029–0.192)	< 0.001
*Age (in years)*		0.003
60–74 vs. 75–84	0.171 (0.050–0.581)	0.005
85–94 vs. 75–84	1.54 (0.894–2.66)	0.119
≥ 95 vs. 75–84	0.468 (0.118–1.86)	0.281
*Mobility*		0.044
Unknown vs. walking independent	0.203 (0.021–1.94)	0.166
Walking with aid vs. walking independent	0.880 (0.469–1.65)	0.689
Wheelchair dependent vs. walking independent	3.02 (1.02–8.95)	0.046
*Presence of cognitive disorder*		0.044
Yes vs. no	1.96 (1.16–3.33)	0.012
Unknown vs no	1.73 (0.226–13.3)	0.597
*Hospitalization one year before ED visit*		0.020
1 vs. none	2.48 (1.28–4.79)	0.007
≥ 2 vs. none	1.67 (0.764–3.63)	0.199

## Discussion

### Summary

Our results show that in less than half of the referred older patients, at least one preference regarding life-sustaining treatments was known by the referring physician at the time of referral to the ED for an acute geriatric assessment. Known preferences always concerned CPR policy, with invasive ventilation and ICU admission being mentioned occasionally. Elderly care physicians were more often aware of their patients’ preferences regarding life-sustaining treatments. There are several possible reasons for this. Firstly, the Dutch *quality framework of nursing home care* [23] states that within 24 h of admission to a nursing home, a (draft) medical plan should be made including treatment limitations regarding CPR policy. Although national guidelines are in place to stimulate GPs to discuss these items with (frail) older people, this is not obligatory [16, 24]. Secondly, elderly care physicians have a regional medical records system in which an on-call physician can access all notes made by colleagues, in contrast to GPs who cannot always access patient medical records during out-of-hours services.

### Strengths and limitations

To our knowledge, we are the first to assess the extent to which older patients’ preferences regarding life-sustaining treatments when referred to the ED for an acute geriatric assessment are known by the referring physician. Moreover, we are the first to study determinants of the presence of these preferences at referral. Previous studies were merely performed in the United States [25–27], were mostly limited to patient-reported outcomes [28–30], or were not performed at referral [31, 32]. A strength is our methodology; daily practice was best reflected by using a direct approach with the geriatrician asking the referring physician whether preferences were known.

We note a number of limitations. First, during the study period, we observed that in about one-third of the referrals, on-call geriatricians did not consistently ask each referrer whether life-sustaining treatment preferences were known. We had to remind the geriatricians that the study procedure was still ongoing. It is likely that in the majority of these cases, the geriatrician simply forgot to ask, due to, for example, a high workload. A second possibility is that the geriatrician forgot to document the answer when it concerned patients of whom the referring physician did not know preferences. This would have led to an overestimation of known preferences, while the extent to which preferences are known is already limited. A third reason may be that the on-call geriatrician did not feel the need to ask about known preferences; they may have been aware of the preferences regarding life-sustaining treatments or they expected that the acute geriatric assessment at the ED would not lead to hospitalization. As we did not collect data on reasons for geriatricians not asking about and/or documenting known preferences, we can only speculate. However, we found selection bias unlikely, and it does not change our conclusion.

Second, this study was performed in the Netherlands, the only country with elderly care medicine as a medical specialty. Therefore, generalizability is limited regarding settings similar to the Dutch healthcare system. However, other countries have systems where each citizen has a GP who functions as a gatekeeper. Future research should examine whether the extent to which preferences were known depends more on the setting or more on the medical specialism of the referring physician.

Third, we did not distinguish whether patients were referred by their own physician or by an on-call physician. In the latter case it is likely that the patients’ preferences would be less known to the on-call GPs, as they may not have had access to the electronic medical records stored in the patients’ own general practice. This in contrast to elderly care physicians who have a regional medical records system in which the on-call physician can access all notes made by colleagues. Moreover, we only asked the referring physician whether preferences were known, and we did not collect information on whether preferences had only been discussed or documented. Both could lead to an over- or underestimation of the known number of preferences. However, in a recent study [33] the percentage of discussing and documenting limitations on life-sustaining treatments was almost equal (73.1 and 70.7% respectively). Additionally, it is important to note that only registered and accessible information can influence decision making by a physician other than the patients’ own physician.

A final limitation is that we focused on preferences regarding life-sustaining treatments, while ACP is a dynamic process that goes beyond this [2]. It is also important to include goals of care [34]. However, especially in an acute setting, all practitioners should be aware of preferences regarding life-sustaining treatments in order to provide more appropriate, desired, and personalized care. Therefore, we focused on those preferences which conform to ACP guidelines in which it is recognized that ADs which concern preferences regarding life-sustaining treatments are important for in-the-moment decision making [1].

### Comparison with existing literature

We show that preferences regarding life-sustaining treatments were known in almost half of the patients being referred. Similar results can be found in the literature. A US cross-sectional study [27] was performed in which they assessed patient-reported completion of ACP and availability of ACP documentation. They characterized completion and availability of ACP among a subset of older patients at an academic ED with an integrated electronic health record. Among study patients, 59% reported having completed some form of ACP: living will 52%, healthcare power of attorney 54%, do not resuscitate 38%, and either medical orders for scope of treatment or physician orders for life-sustaining treatment 6%. In a systematic review which only included US studies, patient-reported AD completion ranged from 21 to 53% [26]. ADs were only available to ED personal in 1–44% of cases. We also show that preferences regarding life-sustaining treatments were known in almost 35% of the patients referred by the GP. These results support a Dutch study where 60% of patients aged ≥75 in general practice had thought about end-of-life treatment preferences, but an ACP conversation with the GP rarely occurred [33]. We also confirm that preferences are mainly limited to CPR policy, invasive ventilation, and ICU admission. A study in Dutch general practices showed that GPs documented CPR policy in 28% and IC policy in 4% of the patients with lung or colon cancer [35].

Our results also show that preferences were more frequently known at repeated ED referral than at first ED referral. A recently published study reports that ED visits trigger a revision of limitations of life-sustaining treatment in most adult palliative patients with solid tumours who died < 3 months after their ED visit. Before the ED visit, limitations on life-sustaining treatments were discussed in 33.8% of the cases, but this increased during or after ED visits to 70.7% [36].

### Implications for research and/or practice

Patient preferences were unknown by the referring physician for more than half of the older patients referred to the ED for an acute geriatric assessment. This has implications for clinical practice in (frail) older people as ACP (including ADs) guides care when a patient is incapacitated or unable to communicate. Without ACP and/or ADs, the patient’s legal representative can be asked about the patients’ preferences which may be experienced as a burden for the legal representative [37]. Moreover, during crises, there is little time to discuss ACP or locate ADs. For that reason, prior knowledge of preferences is valuable, as patients are then more likely to receive desired and appropriate care in line with their preferences and values.

In about half of rehospitalized patients, preferences were still unknown by the referring physician. We hypothesize that this information often gets lost during information transfer between healthcare professionals. Therefore, we recommend that discussed preferences always be explicitly documented, and if possible, communicated by phone and/or referral letters when a patient is transferred between primary and secondary care. Furthermore, we also recommend studying the perspectives of frail older people regarding the timing of ACP conversations and the content of those conversations, in order to contribute to the existing evidence on this topic [38, 39].

As physicians find it difficult to talk about end-of-life treatment preferences, we recommend that they be better trained to initiate ACP conversations; this was shown to be highly effective in a study on ACP by GPs with dementia patients [40]. It appears that barriers to initiate ACP conversations are lack of time, knowledge, and expertise [41], especially when it concerns frail older people [14]. The disease trajectory of frail older patients is characterized by prolonged deterioration and may be cut short by death after an acute event [42]. Therefore, estimating prognosis is difficult, resulting in delays in the conversation on treatment preferences [43], while hospitalization or an unclear prognosis are actually reasons to initiate ACP [44].

GPs and elderly care physicians are both in a position to initiate ACP conservations, as both have a long-term patient relationship and the ability to discuss this in a quiet place with sufficient time and without the need for direct decisions [45, 46]. Preferably, these conversations should not take place at the ED. There are also several non-acute moments at a geriatric department in which ACP could be initiated or continued. A recent ED visit or hospitalization can be a trigger and reference to initiate ACP conversations [47] as referring to this recent situation makes the discussion more tangible. Moreover, geriatricians are experts when it comes to frailty and comorbidity. They possess knowledge that can support GPs and elderly care physicians in these ACP conversations. Therefore, geriatricians can also contribute to a consistent and clear transmural message; ACP should be a shared responsibility of professionals in primary and secondary care.

## Conclusions

To conclude, we found that patient preferences were known by the referring physician in less than half of the older patients referred to the ED for an acute geriatric assessment. When known, these preferences were mainly limited to CPR policy, and occasionally to invasive ventilation and ICU admission. This limited knowledge of preferences may lead to less personalized care with unnecessary futile interventions, unwanted life-prolonging treatments, but also to undertreatment based on ageism. Therefore, ACP needs more attention in daily practice in order to provide more appropriate, desired, and personalized care to older patients in a non-acute setting.

## Supplementary Information

**Additional file 1: Supplementary Table 1.** Known preferences stratified by type of referrer, and patient and demographic characteristics, N (yes/total) (%)

## Data Availability

The datasets used and analysed during the current study are available from the corresponding author on reasonable request.

## Abbreviations

ACP: Advance Care Planning
AD(s): Advance Directive(s)
CCI: Charlson Comorbidity Index
CCU: Coronary Care Unit
CPR: Cardiopulmonary Resuscitation
ED: Emergency Department
GP(s): General Practitioner(s)
ICU: Intensive Care Unit
OR: Odds Ratio
