# Supplementary material for: Life-sustaining treatment preferences in older patients when referred to the emergency department for acute geriatric assessment: a descriptive study in a Dutch hospital
Source: BMC Geriatr. 2021 Jan 14;21:58. doi: 10.1186/s12877-020-02002-y (PMC7807792; doi:10.1186/s12877-020-02002-y)
Supplement: Supplementary file 1 — Additional file 1: Supplementary Table 1. Known preferences stratified by type of referrer, and patient and demographic characteristics, N (yes/total) (%) [file 12877_2020_2002_MOESM1_ESM.docx]

Supplementary table 1. Known preferences stratified by type of referrer, and patient and demographic characteristics, N (yes/total) (%)

|  | ***Preferences known; N yes/total (%)*** | | |
| --- | --- | --- | --- |
|  | ***Elderly Care Physician*** | ***GP*** | ***Total*** |
| ***Age, per category***  *60-74*  *75-84*  *85-94*  *≥ 95 years* | 7/10 (70.0)  23/26 (88.5)  27/28 (96.4)  4/5 (80.0) | 2/22 (9.1)  40/110 (36.4)  53/135 (39.3)  2/12 (16.7) | 9/32(28.1)  63/136 (46.3)  80/163 (49.1)  6/17 (35.3) |
| ***Sex***  *Male*  *Female* | 21/26 (80.8)  40/43 (93.0) | 42/107 (39.3)  55/172 (32.0) | 63/133 (47.4)  95/215 (44.2) |
| ***Living situation***  *Nursing home*  *At home, with:*   - *No home care services* - *Home care services* | 61/69(88.4)  -  -  - | -  97/279 (34.8)  30/99 (30.3)  66/176 (37.5) | 61/69 (88.4)  97/279 (34.8)  30/99 (30.3)  66/176 (37.5) |
| ***Mobility***  *Walking:*   - *Independent* - *With walking aid (walking stick/wheeled walker)*   *Wheelchair dependent* | 2/4 (50.0)  29/32 (90.6)  29/31 (93.5) | 22/66 (33.3)  66/193 (52.0)  8/14 (57.1) | 24/70 (34.3)  95/225 (42.2)  37/45 (82.2) |
| ***Presence of a cognitive disorder***  *Yes*  *No* | 52/60 (86.7)  6/6 (100) | 63/151 (41.7)  33/125 (26.4) | 115/211 (54.5)  39/131 (29.8) |
| ***Charlson comorbidity index (CCI)***  0  1-2  ≥ 3 | 1/1 (100)  28/60 (87.5)  32/36 (88.9) | 9/36 (25.0)  56/160 (35.0)  32/81 (39.5) | 10/37 (27.0)  84/192 (43.8)  64/117 (54.7) |
| ***Number of prescriptions***  0  1-5  6-10  11-15  >15 | -  3/3 (100)  20/22 (90.9)  24/28 (85.7)  12/14 (85.7) | 2/5 (40.0)  23/78 (29.5)  34/114 (29.8)  31/63 (49.2)  7/18 (38.9) | 2/5 (40.0)  26/81 (32.1)  54/136(39.7)  55/91 (60.4)  19/32 (59.4) |
| ***Number of hospitalizations during one year before ED visit***  0  1  ≥ 2 | 43/48 (89.6)  12/13 (92.3)  6/8 (75.0) | 57/195 (29.2)  25/50 (50.0)  15/34 (44.1) | 100/243 (41.2)  37/63 (58.7)  21/42 (50.0) |
| ***Mortality during ED visit or during the subsequent hospitalization***  *No*  *Yes* | 55/62 (88.7)  6/7 (85.7) | 87/262 (33.2)  10/17 (58.8) | 142/324(43.8) 16/24 (66.7) |
